# Inflammatory Processes in Alzheimer’s Disease—Pathomechanism, Diagnosis and Treatment: A Review

**DOI:** 10.3390/ijms24076518

**Published:** 2023-03-30

**Authors:** Bartosz Twarowski, Mariola Herbet

**Affiliations:** Chair and Department of Toxicology, Faculty of Pharmacy, Medical University of Lublin, Jaczewskiego 8b Street, 20-090 Lublin, Poland

**Keywords:** Alzheimer’s disease, inflammatory, neuroinflammation

## Abstract

Alzheimer’s disease is one of the most commonly diagnosed cases of senile dementia in the world. It is an incurable process, most often leading to death. This disease is multifactorial, and one factor of this is inflammation. Numerous mediators secreted by inflammatory cells can cause neuronal degeneration. Neuritis may coexist with other mechanisms of Alzheimer’s disease, contributing to disease progression, and may also directly underlie AD. Although much has been established about the inflammatory processes in the pathogenesis of AD, many aspects remain unexplained. The work is devoted in particular to the pathomechanism of inflammation and its role in diagnosis and treatment. An in-depth and detailed understanding of the pathomechanism of neuroinflammation in Alzheimer’s disease may help in the development of diagnostic methods for early diagnosis and may contribute to the development of new therapeutic strategies for the disease.

## 1. Introduction

In recent years, the demographic situation of highly developed countries in the world has been moving toward an aging population. Among the diseases that are currently most often diagnosed in the elderly, the percentage of diagnoses is related to neurodegenerative diseases, among others. Alzheimer’s disease is one of the most frequently diagnosed cases of senile dementia in the world. It is an incurable process, most often leading to the death of the suffering patient [1]. It was first described by the German physician Alois Alzheimer in 1906. During the pathomorphological examination of the brain of Auguste Deter, who suffered from episodes of memory loss and personality changes before her death, the scientist found the presence of amyloid deposits and massive neuronal degeneration in the collected dissection material and described the observed changes as a serious disease of the cerebral cortex [1,2].

Alzheimer presented his patient’s clinical case on 3 November 1906 at a meeting of the Society of South-West German Neuropsychiatry in Tübingen, describing the disease he discovered as “the disease of forgetfulness” (German “Krankheit des Vergessens”) [3]. The term “Alzheimer’s disease” had already appeared in The Psychiatry Handbook published by Emil Kraepelin in 1910. The name of the disease was officially accepted only at the medical congress in Lausanne in 1967.

Currently, about 55 million people worldwide suffer from Alzheimer’s disease, and this number doubles every 5 years [4]. It is estimated that by 2050 the number of ill patients will increase to approx. 152 million, with the highest increase expected in developing countries [5]. Among developed countries, about 1 in 10 elderly people (65 and over) are affected by Alzheimer’s disease in its early stages, while more than 1/3 of very elderly people (85 and over) may have advanced symptoms and signs of Alzheimer’s disease [6]. It is worth emphasizing that in population studies conducted in European countries, an increase in the percentage of patients with Alzheimer’s disease was shown from 0.6% in the age group of patients aged 65–69 to 22.2% in patients aged 90 and more, which additionally confirms the global trends in the prevalence of Alzheimer’s disease [7].

According to information provided by global reports, in 2020, the estimated costs of Alzheimer’s disease, which included treatment and care for sick patients, amounted to approximately $305 billion, with forecasts showing an increase in expenses to over $1 trillion in 2050 [8].

## 2. AD Pathomechanisms

Despite the fact that almost 116 years have passed since the discovery of Alzheimer’s disease, its pathomechanism is still not fully understood. However, the dynamic progress in medicine and the development of more and more modern research techniques have made it possible to discover macro and microscopic markers that have allowed us to better visualize the nature of the mechanism of development and progression of the disease [9].

### 2.1. Anatomical and Pathomorphological Macroscopic Changes

The first descriptions of anatomical and pathomorphological changes were made by Alois Alzheimer during research carried out in 1906. Macroscopic changes were found in post-mortem examinations of the affected patient’s brain, which are part of the clinical criteria for the diagnosis of the so-called definitive Alzheimer’s disease [9]. An autopsy carried out by a pathologist has allowed the diagnosis of definitive Alzheimer’s disease in nearly 85% of cases examined in forensic centers around the world.

The patient’s brain atrophy of the hippocampus and cortex is a result of the loss of neurons in these areas, which can worsen as the disease progresses and the patient ages [10,11].

In addition, there is a deterioration of cognitive functions in the brain, which is caused by the aforementioned atrophy of the hippocampus and is associated with the accretion of the Tau protein. Structural changes are particularly evident in the amygdale, cingulate cortex, and cortical associative areas of the frontal, temporal. and parietal cortex, but also in the subcortical nuclei [9,12]. The microscopic examinations carried out by pathologists reveal the presence of amyloid deposits and fragments of proteins. The formation of these deposits in the AD brain follows a specific pattern, starting in the pre-olfactory cortex, then extending to the entorhinal cortex, the CA1 region of the hippocampus, and ends in the cortical associative regions where the frontal, parietal, and temporal lobes are particularly affected [13]. The extent and location of plaque formation correlate well with the severity of senile dementia. The result of these changes is the activation of the immune response. Central nervous system (CNS) phagocytes are also activated, i.e., monocytes and tissue macrophages [12].

### 2.2. Amyloid Plaques and Tau Protein

Under the conditions of a healthy organism, β-amyloid is a small, water-soluble peptide. Its formation is the result of splitting fragments of the native amyloid precursor protein (APP), with the participation of α-secretase, β-secretase, and γ-secretase [14,15]. APP is an approximately 770 amino acid glycoprotein that is found in the cell membranes of many cells in the body, including neurons [16]. When the assembly of APP fragments is disturbed, toxic oligopeptides are formed, consisting of 39 to 43 fragments, including protofibrils or fibrils. These, in turn, form deposits that are visible during microscopic examinations [17]. The formation of deposits will not occur if β-amyloid does not have a stable structure, which is related to mutations leading to the destabilization of its structure. The β-amyloid fragments, mainly the Aβ-42 isoform formed by the above-described processes, exhibits cytotoxic properties, in particular towards neurons, which promotes the formation of oxygen radicals that are toxic to nerve cells [18,19]. Their toxicity is mainly based on the dysregulation of calcium homeostasis as a result of lipid disturbances in the cell membranes of neurons, causing their death [20,21,22]. Another component of the plaque formation process is the Tau protein. It is a factor that promotes the process of the specific assembly of the tubulin protein. Tubulin, on the other hand, undergoes polymerization and forms microtubules that shape the intracellular pathway, along which the motor proteins of cells move or take part in cell division, acting as a dividing spindle. In the pathogenesis of Alzheimer’s disease, the formed and deposited neurofibrillary fibers are the result of the hyperphosphorylation of the Tau protein [23]. The neurotoxicity of the Tau protein is based on two main pathways: the loss of physiological function by a normal Tau protein, which causes the destabilization of microtubules, or the gain of function from toxic to neurons, which leads to apoptosis [24]. In their research, many scientists have also proved the correlation between the accumulation of β-amyloid and the aggregation of the Tau protein, which is the final stage of disease pathogenesis [25].

### 2.3. Oxidative Stress

The brain uses about 20% more oxygen than other organs, making it more exposed to reactive oxygen species (ROS) and reactive nitrogen species (RNS) [26,27]. Both ROS and RNS are very unstable structures due to the unpaired electron present within their molecules. In patients with confirmed Alzheimer’s disease, changes related to oxidative damage and neuronal tissue are often observed [28,29]. ROS and RNS interact with neurons due to the fact that they are made of a large amount of polyunsaturated fatty acids. This results in lipid oxidation, the alteration of the redox potential of β-amyloid metal ions, or mitochondrial dysfunction, leading to the apoptosis of nerve cells. In addition, lipid oxidation, as a result of oxidative stress and breaking bonds in DNA molecules, accelerate the aging and death of neurons.

Neuron apoptosis is thus associated with the development of cortical atrophy, which induces the development of Alzheimer’s disease [30]. The peroxidation of polyunsaturated fatty acids results in the formation and accumulation of 4-hydroxy-2,3-none-nal (HNE), malondialdehyde, and F2-isoprostanes. These compounds can stimulate Tau protein hyperphosphorylation and disrupt the calcium homeostasis of neuronal cell membranes, inducing the process of apoptosis in nerve cells [31]. It is also worth noting that both nuclear and mitochondrial DNA can undergo oxidative damage by ROS produced in the brain [32]. The consequence of these actions is the aforementioned breaking of bonds in DNA molecules and between DNA molecules and proteins. Many studies have proven that DNA nucleotide oxidation results in the production of compounds that have been used as markers to determine the extent of changes caused by oxidative stress [32,33,34]. One of the mechanisms of development for Alzheimer’s disease as a result of the presence of oxidative stress is the ROS reaction with glycoproteins. The products of these reactions called advanced glycoxidation end products (AGEs), show significant toxicity to nerve cells. In addition, they may release inflammatory mediators, including IL-1 and TNF-α, inducing neuroinflammation in the brain of a person suffering from Alzheimer’s disease [35]. Moreover, it has been shown that the changes resulting from the activity of oxidative stress in the development of AD can influence the mutation of the physiological Tau protein into the forms of AGEs which are responsible for all the changes described above.

### 2.4. Cholinergic Changes

One of the major neurotransmitters in the human brain is acetylcholine. It is active in the entire cerebral cortex, basal ganglia, and forebrain. This activity is manifested by the participation of acetylcholine in the processes of neuroplasticity caused by the experience acquired during human development; moreover, this neurotransmitter participates in the process of neuronal synchronization, ensuring connectivity between neurons, which determines the appropriate signal transmission [36,37]. One of the first alternative hypotheses for the development of this disease was the cholinergic changes described by scientists in 1976 [38].

This hypothesis can be characterized as atrophy combined with the loss of synaptic connections, resulting in a deficiency in neurotransmission in the brain of an Alzheimer’s patient [7]. The loss of cholinergic conductivity is the degeneration of the Nucleus Basalis of Meynert (NBM): cholinergic neurons and axons that project into the cerebral cortex [39]. The first studies of patients suffering from Alzheimer’s disease showed that changes in cholinergic conductivity, which were already observed in the first stages of the disease, were mainly presynaptic, not postsynaptic [40]. In addition, it has been proven that the changes that occur in nerve conduction in Alzheimer’s disease include nicotinic (ionotropic) receptors and muscarinic (metabotropic) receptors in the cerebral cortex [41]. Early Alzheimer’s disease causes a loss in cholinergic neurons in both the basal nucleus and the cingulate cortex. With disease progression, cholinergic neurons are lost mainly in the basal nucleus in over 90% of the total number of cells in this part of the brain [42]. The result of this process is the decreased binding of cholinergic receptors, which causes the presence of neuropsychiatric symptoms of AD in patients [43]. The process of reduced binding of cholinergic receptors can also occur in healthy elderly people, but it is not associated with the occurrence of Alzheimer’s disease, only with the reduced efficiency of the memory process associated with the natural aging of the brain [44]. It is also worth mentioning that changes consisting of a reduction in the binding of cholinergic receptors in many regions of the brain may constitute a basis for the development of a new generation of molecular drugs used in patients with Alzheimer’s disease [45].

### 2.5. Genetic Changes

So far, no unequivocal reasons for considering genetic changes as the main and most important factor in the development of Alzheimer’s disease have been demonstrated [46]. Mutations of the genes encoding the amyloid precursor protein and mutations of the ε4 allele of apolipoprotein E (ApoE), which have been identified as the most dangerous genetic risk factor associated with the development of AD at the time of writing this work, rarely occur [47]. On average, mutations of the ε4 allele occur in about one-fifth of patients with Alzheimer’s disease, which is about 65% of all patients affected by this disease. In carriers of this mutation, the risk of developing the disease increases almost threefold. The mechanisms of induction for genetic changes within the ApoE allele have not yet been thoroughly investigated and described; however, the studies conducted so far show their relationship with a decrease in β-amyloid clearance, which may lead to its formation in the brain and result in the development of AD [48]. Additionally, the relationship between the presence of two preselin alleles (PSEN 1 and PSEN 2) in patients with Alzheimer’s disease and patients predisposed to Alzheimer’s disease should also be mentioned. The frequency of these alleles in AD patients is relatively low [49]. Mutations in the PSEN1 allele on chromosome 14 occur in 18–50% of cases; mutations in the PSEN2 allele on chromosome 1 are less common than mutations in PSEN1 [50].

### 2.6. Mitochondrial Dysfunction

During the development of Alzheimer’s disease, mitochondrial dysfunction is associated with the impairment of mechanisms that protect the mitochondria against the action of reactive oxygen species (ROS) [51,52]. Many hypotheses, supported by appropriate studies, have proven that two out of four mitochondrial complexes are alternated: complex I and complex II are the most damaged as a result of ROS action. Under physiological conditions, the mitochondria were equipped with antioxidant systems, including cytochrome c oxidase [53]. It has been shown that when the body develops AD, the activity of cytochrome c oxidase in the hippocampus is significantly reduced. However, it is not only oxidative stress and ROS defective defense that can damage mitochondria in AD patients. It is worth adding that β-amyloid oligomers can enter the mitochondria in two ways: due to a higher expression of ER membrane proteins associated with the mitochondrial membrane and due to transport through the outer membrane complex [54]. Their accumulation in the inner membrane of CNS mitochondria disturbs the functioning of components in the electron transport chain, which additionally increases the production of ROS and drives the pathomechanism described earlier; this is related to impaired antioxidant defense and the formation of oxidative stress [55,56].

## 3. Inflammatory Processes in Alzheimer’s Disease

A large amount of research and scientific studies indicate inflammatory processes as the mechanism most often present in the development of Alzheimer’s disease [57]. A set of many cells, including astrocytes, microglia cells, and proteins such as cytokines and chemokines, cause neurinflammation, damages the environment surrounding neurons, leading to their damage, and thus contribute to the development of oxidative stress or leads to cell apoptosis, which causes the appearance of symptoms that are indicative of Alzheimer’s disease [58].

The inflammatory process is an expression of a specific, targeted, and enhanced immune, biochemical, and hematological response that can be triggered at the local or systemic level [59]. It is a process that is a reaction to the damage to tissue or organ structures, which, in particular, is designed to neutralize the damaging factor and stimulate processes that enable the restoration of the original state of the organism. The presence of neuroinflammation in the course of AD was described nearly 33 years ago [60,61]. However, despite knowledge about the components of this process and the effects on the AD patient, the exact mechanisms of its formation and the role of neuroinflammation have still not been sufficiently elucidated [62]. By adopting a strictly biological and medical approach, it was possible to determine the similarity of the inflammatory process in AD to classical inflammation in terms of inflammatory factors, which are often the same in both types of reaction [63]. The most important substances exerting a pro-inflammatory effect on the brain and the whole organism include, among others: some cytokines (e.g., IL-1β, IL-6, IL-18, TNF-α, IFN-γ), chemokines (e.g., CCL2, CCL3, CXCL8), complement components (e.g., C1q, C5), transcription factors (including, e.g., NF-κB), peptides (e.g., bradykinin), enzymes (e.g., COX-2, iNOS, LOX), and coagulation factors (including, e.g., PAF- platelet-activating factor) [64,65].

The opposite effect is exerted by lipoxins (including, for example, LXA4, RvE1) and some cytokines (such as IL-10, IL-37, TGF-β) [64]. In the classic type of inflammatory reaction, the process is induced by increasing the secretion of pro-inflammatory mediators and reducing the secretion of anti-inflammatory substances, while extinguishing the process is the opposite: the secretion of pro-inflammatory substances decreases as anti-inflammatory substances increase [64]. However, the activity of inflammatory factors differs between the neuroinflammation and inflammation that develops outside the brain [66].

**Figure 1:**
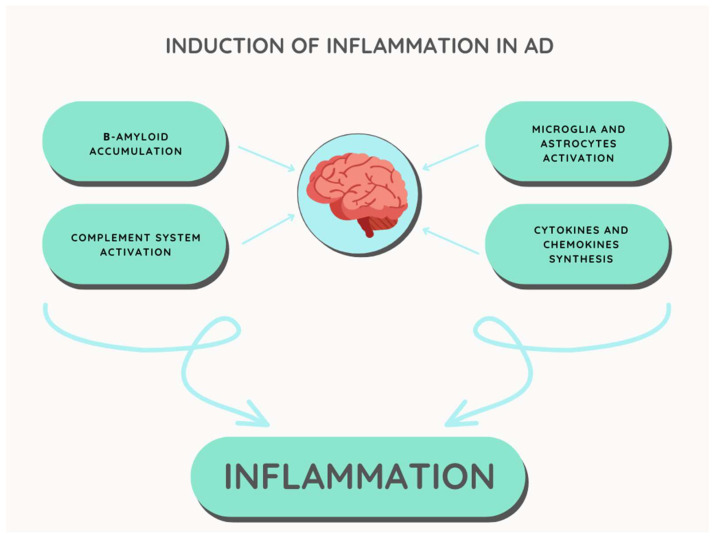
The primary inducers of inflammation in Alzheimer’s Disease.

Neurinflammation produces an effect that is desired and beneficial in the course of the disease, i.e., it activates the immune response in the form of the activation of phagocytic processes that are carried out by both astrocytes and microglial cells in order to eliminate potential pathogens and remove their debris [67]. The aforementioned dual nature of neuroinflammation becomes apparent when we observe the negative effects of this process, causing harmful consequences for neurons in the patient’s brain [12]. In each type of inflammatory reaction, the moment that marks the end of its duration is very important. The inflammation response is muted when particles of the inflammation-stimulating pathogen are removed [68]. It is an active process that is supervised by specialized pro-solving mediators acting as regulators [11,12]. Numerous studies have demonstrated a reduced concentration of neuroinflammation suppression regulators, which may be related to β-amyloid deposits present in the brain of AD patients, driving the inflammation process and causing its permanent maintenance. However, it is worth tracing the genesis of the neuroinflammation process [69,70] (Figure 1).

### 3.1. β-Amyloid Amyloid Hypothesis

There is a theory that β-amyloid itself induces the inflammatory process due to its accumulation in the brain and the ability to bind the C1 component of the complement system, inducing neuroinflammation [71]. It can therefore stimulate native CNS immune cells to induce an inflammatory reaction against the accumulating β-amyloid that forms the deposits surrounding these cells. In order for the complement system to be activated, β-amyloid must be properly fibrillated [72].

This is confirmed by numerous discoveries in the field of clinical pathomorphology, for which immunohistochemical studies were used and carried out on the post-mortem material from AD patients [73]. There are diffuse and classic plaques in the brain of a patient suffering from AD. Diffuse plaques cannot bind to activated microglia cells, while classical plaques can [74,75]. The ongoing inflammatory process in the patient’s body is particularly visible on classic plaques due to the presence of connections with activated microglia. The chronic nature of neuroinflammation can thus be confirmed. Studies supporting this theory have shown that the inflammatory process is especially induced by β-amyloid, which is deposited extracellularly in the brain of AD patients [76].

The significance of the formation of β-amyloid deposits in the brain of Alzheimer’s patients has become a subject of research by scientists in many countries around the world. Hommet et al., demonstrated the link between amyloid deposition and the induction of neuroinflammation by conducting a cross-sectional analysis of original studies that deal with the molecular imaging of the inflammatory process in Alzheimer’s disease, with a particular focus on visualizing amyloid plaques that constitute an inflammatory stimulus. Common features of all the studies that the authors considered in selecting the studies for analysis included the characteristics of the study sample, the general description of the study, radionuclides used as markers, and the results and conclusions related to the involvement of the amyloid deposition process and the induction of neuroinflammation in AD [77]. A clinical study was conducted using advances in nuclear medicine, specifically the positron emission tomography (PET) method. A set of radiolabels was used to visualize amyloid deposits: carbon-11-labeled isoquinolinecarboxamide PK-11195, which was used to quantify the inflammatory process; Pittsburgh compound B, also carbon-11-labeled, with which the amyloid load could be determined; and F18 fluorodeoxyglucose (F18-FDG), which is routinely used in nuclear medicine [77]. The clinical results were as follows: one of the first radio markers to reflect the severity of amyloid plaque formation in AD patients was Pittsburgh’s carbon-11-labeled compound B. The accumulation of this radiolabel was associated with an increase in the mass of deposits. However, this tracer’s short half-life of 20 min severely limits its use in clinical practice. Other radionuclides, created on the basis of fluorine-18 isotopes, have shown similar results to C11-PiB, making it possible to effectively identify the formation of amyloid deposits and thus distinguish between Alzheimer’s patients and healthy patients. In addition, Hommet et al., cited an example authored by the clinical team formed by Yokokura et al., which recorded a significant increase in the uptake of carbon-11-labeled PK-11195 in AD patients compared to healthy patients. In addition, they found that AD patients were characterized by a reduced uptake of F18-FDG in the anterior and posterior regions of the cingulate cortex in the early stages of the disease when the synthesis of amyloid plaques was less intense. This is not unique to Hommet et al., as they report that Wiley et al., in their study, found that the retention of carbon-11-labeled PK-11195 did not differ between sick and healthy patients regardless of the presence of amyloid deposits, while opposite results were obtained by Edison et al., who found an increase in PK-11195 retention in Alzheimer’s patients. This shows the great complexity of neuroinflammatory processes and the fact that the study of β-amyloid and its deposition processes can resolve many of the not fully understood facts related to the occurrence of neuroinflammation in Alzheimer’s disease [77]. The development of Alzheimer’s disease on an inflammatory basis spurred by the deposition of β-amyloid deposits may provide a starting point in the development of new treatment strategies for AD. Tautou et al., conducted clinical trials of the polyamine compound PEL24-199 using APP/PS1 transgenic mice, a model of Alzheimer’s disease. Increased concentrations of β-amyloid can cause damage associated with oxidative stress and inflammation and can up-regulate the levels and phosphorylation of the Tau protein, which consequently results in, for example, the formation of neurofibrillary tangles and the malfunctioning of neural synapses or neuronal death [78]. The authors hypothesized that a therapeutic approach that targeted amyloid deposits could be an effective way to halt the progression of the disease and, in the future, may even lay the groundwork for a complete cure for Alzheimer’s patients. This was also confirmed by the research conducted. The tested compound, which had β-amyloid as its target, effectively abolished the process of neuro degradation caused by neuroinflammatory mechanisms. This shows that the process of accumulation of β-amyloid deposits is one of the many important causes of the development of neuroinflammation [78].

### 3.2. Microglia and Astrocytes

Every human brain has its own immune cells, which are microglia cells. Microglia make up 10% to 15% of all cells found in the brain. In health conditions, microglial cells are responsible for many important functions ensuring the proper functioning of the CNS [79]. Among the many tasks they perform, the following can be distinguished: neurogenesis, the maintenance of nerve conduction, and the regulation of cognitive functions; they are also a component of immune surveillance. The latter function is of particular importance when the patient begins to develop Alzheimer’s disease. As with other phagocytic cells in the body, this role is to remove pathogens entering the CNS and remove cell debris from the neuronal environment [79,80,81]. In addition, microglia may be phagocytosed in relation to the neurons themselves when abnormalities related to their functioning are detected or damaged in some way [73]. It is very important then that the microglia function properly; otherwise, it may cause CNS intoxication through unremoved harmful particles, including, for example, free radicals that cause oxidative damage within the brain tissue [82]. Apart from phagocytosis, an important role of microglia is also the modification of synapses, including creating new connections with them or removing them [82]. As the brain develops, microglial cells form short connections with synapses, which remove weak or unnecessary or underdeveloped synaptic connections. It is an essential element in maintaining a normal neural network and connections between synapses in the CNS [83]. Many studies have proven the heterogeneity of this group of cells, depending on which region of the CNS they reside in [84]. In general, the number of microglial cells remains the same from the postnatal period to old age due to the simultaneous processes of proliferation and apoptosis. Moreover, microglia were originally considered to be “resting” type cells. Meanwhile, they are very dynamic, patrolling the brain within 24 h, thus supporting the proper functioning and health of neurons. If, on the other hand, pathogens or trauma are detected, the microglia are activated. In Alzheimer’s disease, microglia activation occurs when β-amyloid deposits build up. Then, microglial cells proliferate, which increases their number and size, and is proportional to the size of deposits in a given region of the brain of an AD patient [84,85]. Subsequently, the deposits are surrounded by microglia cells, and the extent of this process depends on the distance from the microglia [85]. It is worth emphasizing that numerous studies by scientists dealing with the topic of neuroinflammation in AD have proven that the activation of microglial cells is responsible for its triggering, which occurs much earlier than the clinical manifestation of Alzheimer’s disease, which emphasizes the immune potential of these cells, additionally proving the significant role of these cells in the inflammatory process and the course of AD [82].

The fact that activated microglia cells are directly involved in inflammatory processes has led researchers to better understand the mechanisms of action of these cells on the inflammatory microenvironment of the brain of an AD patient. The pro-inflammatory effects of microglia cells and astrocytes are primarily associated with the formation of amyloid deposits. Temviriyanukul et al., investigated how the anti-inflammatory activity of the Kaempferia parviflora extract found that reducing β-amyloid toxicity and inhibiting the excessive activation of microglia may benefit AD patients. The bioactive fractions of these extracts reduced the activity of microglia and the substances they produced, which include reactive oxygen species (ROS) that are responsible for the induction of oxidative stress in neurons [86]. The authors emphasized that neuronal damage on inflammatory grounds is often caused by excessive phagocytosis carried out by microglia cells. The excessive accumulation of β-amyloid and the formation of deposits causes the hyperactivation of microglia cells and exacerbates the negative effects of this process on neurons, thus causing the progression of neurodegenerative changes in the brains of sick patients and contributing to the deterioration of their overall health. In addition to highlighting the role of potential therapeutic candidates for neuroinflammation in AD patients, the study has opened the way to an even broader understanding of the role of microglia as an inflammation inducer in AD; however, a full understanding of the role of microglia as a cellular inflammatory factor requires detailed research [86]. Huang et al., came to similar conclusions, identifying microglia as a factor for which understanding its function and activation can help determine how to neutralize inflammation in Alzheimer’s patients [87]. The excessive phagocytosis of amyloid plaques by microglia cells has been shown to play an important role in the penetration of β-amyloid into healthy brain regions of AD patients. In vitro studies conducted by the authors showed that, along with the penetration of β-amyloid into healthy regions of neural tissue, microglia furthermore produce a large amount of pro-inflammatory cytokines such as IL-6, which further causes the inflammatory involvement of healthy neurons in AD patients [87].

Another cellular component of the neuroinflammation process is astrocytes [88,89]. Similar to microglia, astrocytes perform many functions to maintain structural and functional homeostasis in the CNS. However, when it comes to immunological processes, scientists do not fully understand the role of these cells in the defense creations of CNS [90]. Studies conducted in mouse models have proven the high immunological and genetic reactivity of astrocytes. They form clusters at the site of the β-amyloid deposition, often creating barriers for microglial cells, which prevents the removal of deposits by microglial cells [91]. In addition, Das et al., found that the astrocyte response to inflammation-related injury in AD was accompanied by increased protein synthesis and degradation. Systems, including the ubiquitin-proteasome system, are activated, which leads to an increase in chaperones, ubiquitin igases, proteasome subunits, and autophagy, which leads to the removal of damaged neurons from the area of injury [92].

### 3.3. Complement System

The inflammatory process that the above-described cell mediators induce in the brain of a patient suffering from Alzheimer’s disease may take many pathways due to the multitude of other factors that are capable of causing it [93]. As in the case of the classic inflammatory reaction, the complement system shows significant pro-inflammatory activity in neuroinflammation, and its components play a special role in the pathogenesis of AD [17]. Complement activation can follow two paths: classical and alternative [93]. If complement activation occurs via the classical pathway, nearly 20 components are involved in this process, and they are activated one after another in many reactions of alternating splitting, hydrolysis, and the reassembly of components. In these processes, apart from the complement component, antibodies may play the role of modulators in the activation of individual complement components [94]. Components that cleave, hydrolyze, and reassemble during activation can form membrane complexes. From the perspective of the induced inflammatory process, the C4/C2 complex, called C3 convertase, is of significant importance due to a high ability to cleave many molecules of the C3 component into C3a and C3b fragments, which, by reuniting with C3 convertase, forms other components, including C5, which it then cleaves to C5a and C5b. C5b, together with C6, C7, C8, and the C9 components, form another important component from the perspective of induced neuroinflammation. The C5b-9 com-ponent complex, otherwise known as the membrane attack complex (MAC), has a pro-inflammatory effect [94,95]. This complex accumulates on the surface of the cell membrane of the target cell, creating a channel that penetrates through the membrane structure. This facilitates the diffusion of ions from inside the cell and into it, which disturbs its ionic balance (especially calcium ion management), leading to the targeting of cell lysis [96].

It is assumed that the presence of β-amyloid deposits is a stimulus that activates the classical complement pathway, for which the presence of antibodies is not required [97]. The binding of β-amyloid by complement takes place through the interaction of the cationic chains of the collagen-like end of the C1q component and the anionic free chains at the N-terminus of β-amyloid [98]. Additionally, amyloid P or the C-reactive protein can also bind to the C1q component, which activates the classical complement pathway [99]. However, it is not only the presence of plaques in the brain of Alzheimer’s patients that can cause complement activation in a classical way [100]. It binds to the same site on the C1 component as β-amyloid. Likewise, neuro filaments, oligodendrocyte myelin glycoprotein, and other myelin-derived proteins can all contribute to complement activation in the same way [101]. It is worth noting that there are numerous factors that can activate the classical pathway of the complement system, which is a significant driving force behind the ongoing process of neuroinflammation in the brain of a person suffering from Alzheimer’s disease [102]. Complement activation may also occur in an alternative way. This process, unlike classic complement activation, is based mainly on the interaction of individual components with β-amyloid deposits [103]. This is a highly specific reaction, which is further supported by the fact that molecules showing structural and functional similarity to β-amyloid do not activate the alternative complement pathway [103]. A characteristic feature of the alternative way of complement activation is the presence of Aβ complexes with fragments of the C3 component [102]. It is worth noting that fibrillar β-amyloid leads to the formation of the C5a component, which then, together with C6, C7, C8 and C9, forms the MAC membrane attack complex, which intensifies the induction of the inflammatory process in AD [104].

### 3.4. Cytokines

One of the substances showing pro-inflammatory activities is cytokines. The cytokine that is abundant in the AD brain is IL-1β. It is produced by activated microglial cells, especially in the early stages of the disease, where there is significant β-amyloid deposition and plaque and plaque formation. In particular, its presence can be detected by immunochemical methods in post-mortem studies of brain tissue, where it is mainly seen in diffuse plaques. These methods additionally confirm the aforementioned fact that IL-1β is a regulator of the early formation of β-amyloid deposits, as the intensity of the staining of the microglia producing it weakens with microglia proliferation and plaque development [105]. It should be noted that IL-1β is involved in the synthesis of β-amyloid because it acts as a regulator of APP synthesis [106]. Moreover, the intermediate forms of APP activate microglia and cause the excessive secretion of IL-1β, which, in turn, stimulates astrocytes, causing the secretion of pro-inflammatory substances [107].

In addition, IL-1β overexpresses S100β, an activated astrocyte cytokine that is involved in the neuronal growth response and is present at high levels in Alzheimer’s patients. S100β in Alzheimer’s disease can cause neuronal dystrophy within amyloid deposits [108]. This is an example of the fact that both IL-1β overexpression and the resulting S100β overexpression result in significant neuronal dystrophy and the accumulation of β-amyloid deposits [109]. It is also important that IL-1β can increase the activity of acetylcholinesterase by influencing mRNA expression, which increases the dysfunction of the cholinergic system in the pathogenesis of Alzheimer’s disease [86]. Another cytokine that is important for the development of neuroinflammation in Alzheimer’s disease is IL-6 [110]. In addition to immune and inflammatory reactions, IL-6 influences the differentiation and growth of nerve cells during the growth and development of the CNS and CNS. Therefore, in a normally functioning adult organism, its concentration is very low, and when it grows, it indicates a developing pathology. Virtually all of the cellular components of AD neuroinflammation are capable of producing IL-6. In general, the overexpression of IL-6 is deleterious and may contribute to the induction of CNS dysfunction [110]. Moreover, IL-6 causes the release of the acute phase protein, increases the permeability of blood vessels, and activates CNS phagocytes, further intensifying the inflammatory process. The long-term effect of IL-6 indicates the existence of a chronic inflammatory process in the brain of an AD patient [111].

By performing the immunohistochemical staining of material collected from patients suffering from AD, the presence of IL-6 in a high concentration was shown mainly in diffuse plaques, which suggests that an increase in IL-6 concentration was observed before neuronal degenerative damage occurred [110,111]. As West et al., found, the pro-inflammatory cytokines IL-6 and IFN-α are capable of inducing neuroinflammation in AD patients, mainly through the continuous production of these cytokines by activated microglia cells, which are stimulated by inflammatory stimuli such as, but not limited to the formation of amyloid deposits [112]. In addition, the authors of the study showed that IL-6 increased the number of microglia cells compared to IFN-α, which did not cause a significant increase in the number of microglia cells. Thus, pro-inflammatory cytokines not only promoted an increase in the phagocytosis processes carried out by native CNS phagocytes but additionally caused an increase in the number of microglia cells and astrocytes, which are locally responsible for these processes [112]. Moreover, researchers found that IL-6 and IFN-α had an important function in immunohistochemical diagnosis due to the increased specificity of the tomato lectin labeling of activated microglia cells producing these pro-inflammatory factors, which increases the sensitivity of diagnostic methods and contributes to the efficiency of diagnosis of inflammatory processes in AD [112]. Changes in the concentrations of pro-inflammatory cytokines in neuroinflammation may also be related to the number of receptors with which these cytokines bind. Haddick et al., analyzed cerebrospinal fluid samples to show a link between IL-6 activity in Alzheimer’s patients and the number of receptors for this cytokine [113]. The main source of their receptors for IL-6 is microglia cells. Studies have shown that in the course of Alzheimer’s disease, cells that are sensitive to IL-6 shed from their surface the receptors that are responsible for binding this cytokine, leading to a decrease in its activity and abolition of its pro-inflammatory effect on neurons in AD patients [113].

### 3.5. Cyclooxygenase Activity

Neuroinflammation in AD may also be related to cyclooxygenase activity. It is an enzyme that catalyzes the synthesis of prostaglandins from arachidonates. Prostaglandins are irreversibly involved in the inflammation process; hence, their presence in the inflammatory reaction in the brain during Alzheimer’s disease is evident. In AD neuroinflammation, COX-2 levels are elevated and often reflect β-amyloid levels [114]. This is mainly seen in the hippocampus and is often associated with neuronal degradation in the AD patient’s brain. Additionally, elevated COX-2 levels can be detected in the frontal cortex of an AD patient. Increasing the concentration of cyclooxygenase may additionally affect the mechanisms that trigger the inflammatory reaction by many other factors that have been described earlier in this study, which include, for example, the inflammatory activity of cytokines [115]. COX can also cause the formation of free radicals, which contributes to the death of neurons from oxidative stress and other oxidative damage, which can lead to inflammation. Finally, the mere elevation of COX-2 levels can damage the neuron, resulting in neuroinflammation, and expose others to other damaging factors present in Alzheimer’s disease [116]. Inflammation in AD can also change from acute to chronic in nature [117]. When the activity of inflammatory factors, including cells and their secreted cytokines, begins to persist for a long time (lasting more than a week and reaching many weeks), we can talk about the chronicity of neuroinflammation [118]. The effects of this process are even more difficult to heal, worsening neurodegeneration and intensifying neuronal apoptosis, which, in turn, exacerbates the symptoms of inflammatory AD [119]. However, it should be emphasized that neuroinflammation is not a specific process, and it is not possible to clearly define the factor that causes it [120]. Many different factors can act independently, inducing the inflammatory process, or they can complement each other, intensifying the effects of developing the inflammatory process; therefore, a more detailed understanding of the factors causing inflammation in AD and the mechanisms by which the described factors work is important from the point of view of a clinical approach to the diagnosis and treatment of neuroinflammation itself and AD as a whole [121].

## 4. Diagnostics of the Inflammatory Process in AD

It is very difficult to diagnose the disease at an early stage of AD development; therefore, the diagnosis of AD-related disorders requires the extensive experience of clinicians managing the patient. The diagnostics of inflammatory conditions that appear in AD is particularly important because, as previously mentioned, it is now believed that inflammation is one of the factors that is particularly responsible for its development [122]. The diagnosis of inflammation in AD is a process involving many branches of medicine, including laboratory medicine, immunochemistry, clinical biochemistry, clinical immunology, hematology, anatomy, histology, and pathomorphology. It can be divided into two basic parts: basic or early, which includes basic laboratory tests often performed routinely by many patients and specialists, or late, which may include the following diagnostics: immunological, including flow cytometry, radiological and imaging diagnostics, with brain imaging of AD patients and pathological diagnostics, which include, among others immunohistochemical reactions and anatomopathological diagnostics, autopsies performed after the patient’s death [123]. Laboratory medicine plays a very important role in the process of diagnosing inflammation and AD itself at an early stage of development [124]. Within it, clinical biochemistry, hematology, and specialized immunological tests are mainly used. The laboratory diagnostics of neuronitis is primarily the determination of the concentration of individual inflammatory factors, the determination of the concentration of characteristic markers of inflammation, increasing in the course of neuroinflammation, and the morphological assessment and immunological phenotyping of inflammatory cells [125]. The characteristic markers of inflammation, including neuroinflammation, that increase when the inflammatory response is triggered include but are not limited to, acute-phase proteins [126]. Acute-phase proteins are proteins that increase the concentration in response to inflammation processes in the body. Numerous studies have shown that inflammatory mediators can affect the concentration of individual acute-phase proteins. Based on this, acute-phase proteins were divided into positive and negative [127,128]. The positive acute-phase proteins, the concentration of which increased in acute-phase reactions, include, among others, the C-reactive protein, α1-antitrypsin, α1-antichymotrypsin, haptoglobin, ceruloplasmin, plasminogen, fibrinogen, procalcitonin, and complement C3 and C4 components [128]. The negative acute phase proteins, the concentration of which decreases in acute phase reactions, include albumin, prealbumin, transferrin, and antithrombin [127].

### 4.1. Determination of C-Reactive Protein Concentration

Among the acute phase proteins, the C-reactive protein (CRP) is the most commonly used in diagnostics. During neuroinflammation, IL-6 is released in large amounts by stimulated immune cells, which stimulates the synthesis of CRP by liver cells [129]. We observed an acute increase in protein within 6–8 h from the action of the damaging factor. Physiologically, the concentration of CRP did not exceed 5 mg/l; however, when the concentration value exceeds 10 mg/L, then it can be considered that the body of the patient with AD develops inflammation [130]. A further drastic increase in this parameter may result in an almost 1000-fold increase in the level. It is worth emphasizing, however, that CRP is the most common marker of an acute condition, so if a patient with Alzheimer’s disease changes to chronic, this marker will not be useful because the concentration of CRP usually normalizes within 24 h [131]. Nevertheless, the determination of the CRP concentration remains one of the most important determinations in the biochemical diagnostics of inflammations, including neuroinflammation in AD [132].

### 4.2. Determination of Pro-Inflammatory Cytokines Concentration and Complement System Activity

In Alzheimer’s disease, the diagnostic culmination point is the determination of pro-inflammatory cytokine levels [133]. Among them, the concentration of interleukin 1, interleukin 6, and TNF-α are measured, which best reflects the picture of neuroinflammation in the brain of a patient with AD [134]. In patients with Alzheimer’s disease, an increase in IL-1 levels was observed [135]. This is associated with the accumulation of β-amyloid, the formation of neurofibrillary fibers, and the phosphorylation of the tau protein [136,137]. Additionally, the amyloid precursor protein increases the concentration of IL-1, which influences the activity of AChE, resulting in the development of AD based on the cholinergic hypothesis described above [138,139]. However, the applied methods are imperfect and require corrections due to the fact that the detected IL-1 concentration in some patients is reduced compared to the healthy samples [140]. IL-6 levels may be elevated due to the accumulation of β-amyloid as deposits in the brain of AD patients early in the process. Additionally, this concentration correlates with the concentration of Il-6 measured in the blood of AD patients. Another important cytokine, determined from the point of view of the diagnosis of inflammation in AD, is TNF-α—a necrotic factor in neoplasms [141,142]. TNF-α is secreted during both acute and chronic inflammation and is strongly associated with a loss in cognitive function and memory deterioration during the development of the disease in a patient due to neuronal apoptosis. In neuroinflammation, TNF-α levels remain elevated in both the serum of AD patients and those with CSF donation. Cerebrospinal fluid samples show a nearly 25-fold increase in concentration compared to physiological CSF [143].

This proves that the level of TNF-α can vary individually, and, despite the general tendency of the increase in the concentration of this parameter in AD, some may experience a decrease in the concentration of TNF-α [129]. IL-6 or TNF-α are often determined by methods of immunological diagnosis [144,145]. The determinations are performed on the basis of ELISA methods, i.e., the measurement of the cytokine level in the serum or in the supernatant obtained after the culture of inflammatory cells with an enzyme immunoassay; ELISPOT methods, i.e., the measurement of the cytokine level in inflammatory cells or the basis of methods measuring the concentration of cytokine mRNA, such as the real-time PCR or northern-blot [146]. In addition, AD patients show an increase in IL-10 levels, as well as an increase in IL-8 levels, as Hu et al., found in their study [147]. The diagnosis of neuroinflammation in AD has been also observed with an increase in IL-18, IL-33, and IL-12, as demonstrated by Jensen and colleagues by measuring the levels of these inflammatory factors in a mouse model [148]. Additionally, the diagnosis of neuroinflammation in AD can be carried out by examining the components of the complement system. The decrease in the concentration of individual components directly proved the activation of this system, as a result of which the factors fell out of circulation due to their binding in the inflammatory focus of people with Alzheimer’s disease [149]. The methods by which the activity of the complement system is measured are similar to those used in the determination of cytokines. Enumerated methods include ELISA and ELISPOT immunological methods, real-time PCR, or the determination of activity and concentration of individual complement components [150].

### 4.3. Histological and Pathomorfological Methods

The diagnosis of neuroinflammation also includes histology and pathomorphology. Among the methods of histopathological diagnostics, one should, first of all, mention immunohistochemical reactions, which are mainly used when clinicians confirm the presence of neuritis in AD and Alzheimer’s disease itself [140]. Immunohistochemical reactions are methods based on the classic immune reaction between an antigen and an antibody, which can result in the formation of antigen–antibody immune complexes. As a result, these are techniques that are characterized by the very high sensitivity, specificity, and repeatability of the obtained results [151,152]. The reaction is revealed through the use of fluorochromes or enzymes with which the antibodies used in this method are labeled. There are two variants of this method: direct, where the labeled antibody binds directly to the desired antigen, and indirect, where first the antigen is bound by the primary antibody, and then the resulting complex is detected by binding to the labeled antibody [153]. However, determinations are carried out more frequently using the indirect method [151].

Immune reactions are mainly used to detect β-amyloid deposits, the Tau protein, ubiquitin and neurofilaments [154,155]. The detection of β-amyloid deposits is also particularly important from the perspective of the diagnosis of neuroinflammation [156]. This is possible thanks to the use of immunohistochemical staining with thioflavin S [157]. Around them, microglial cells that are activated by inflammation accumulate and then join together, which, after detecting amyloid deposits, can also be visualized [158]. In immunohistochemical tests, activated microglia are stained with leptins, which stain the cytoplasm of cells brown, and hematoxylin, which stains the cell nucleus blue [140].

### 4.4. Genetic Diagnostics

From a clinical point of view, the genetic diagnostics of inflammation that develops in the course of Alzheimer’s disease is also important [159]. These are not first-line tests but specialist tests that can confirm ongoing neuroinflammation [50]. Genetic diagnostics include, among others, the detection of polymorphisms of genes related to inflammatory complement activation, including CLU, CR1, SERPINA3, CRP, C2, CFH, C3, and C4. The polymorphism of their genes and the identified mutations of these genes may be a helpful complement to the diagnosis of inflammation in AD, significantly facilitating the work of clinicians [160]. Interdisciplinary research used in the diagnosis of inflammation should mutually confirm these results and complement each other [161]. An example can be an algorithm in which the detection of β-amyloid deposits in histopathological tests and then the identification of activated microglia around them (with the simultaneous detection of an increased concentration of inflammatory cytokines, which can additionally be confirmed by specialist genetic diagnostics), may indicate the development of neuroinflammation in a patient with AD [162]. The given example also shows how important the cooperation of the clinical team is, including doctors of various specialties or laboratory diagnosticians, in order to make a precise and clear diagnosis, enabling the correct determination of the condition of a patient suffering from AD and the selection of an appropriate treatment method [163].

## 5. Treatment of Inflammation in AD

**Figure 2:**
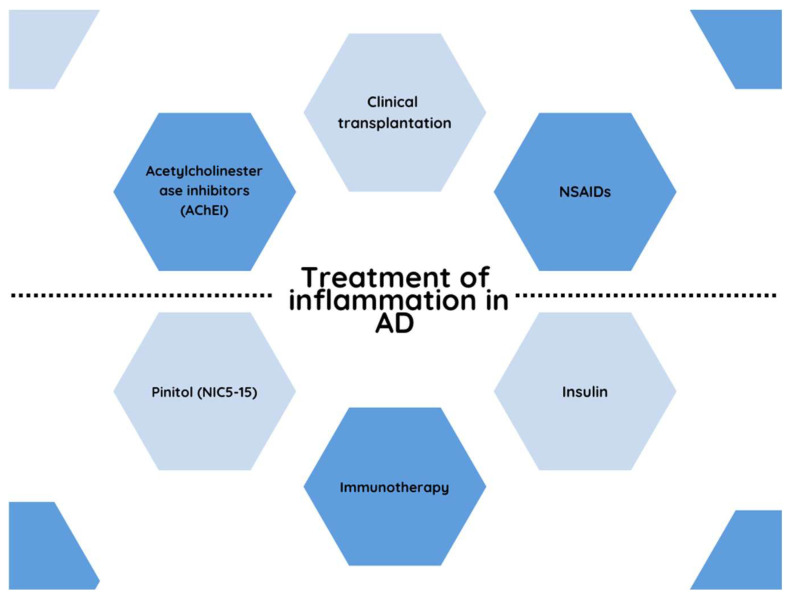
Current treatment strategies used in therapy of inflammation in Alzheimer’s disease.

Alzheimer’s disease undoubtedly presents a clinical challenge for the medical team caring for sick patients [164]. After understanding the mechanisms leading to the development of the disease and ordering appropriate diagnostics to determine the patient’s condition, it is time to select an appropriate treatment method [165]. The treatment of Alzheimer’s disease generally aims to delay the development and progression of the disease [166]. The aim of such pharmacotherapy is to improve the general health of the patient and their well-being and to increase the comfort of functioning in everyday life. It should be emphasized that the effect of this type of pharmacotherapy is limited in time [167]. According to the current state of knowledge, drugs used mainly in the treatment of AD may, inter alia, affect the activity of enzymes involved in the synthesis of abnormal forms of proteins, which are important in the pathogenesis of AD or in the improvement of cholinergic neurotransmission [168] (Figure 2).

Acetylcholinesterase inhibitors (AChEI) are widely used today. Acetylcholinesterase is an enzyme that breaks down the neurotransmitter acetylcholine at cholinergic synapses [169]. The prevention of the breakdown of acetylcholine increases cholinergic neurotransmission, which at the same time delays the development of AD. In European Union countries, including Poland, the acetylcholinesterase inhibitors available for AD therapy are donepezil, rivastigmine, and galantamine [170,171]. The latter, due to a lack of reimbursement, is practically not used at all [40]. In addition, it is believed that AChEIs may have a stabilizing effect on existing Alzheimer’s disease, causing a slower process of cognitive deterioration, which can improve the quality of life of sick patients [172]. It is also worth mentioning that the use of AChEIs delays the placement of AD patients in specialized nursing homes and significantly reduces the time spent by medical staff on a patient treated with AChEIs compared to a patient not treated with AChEIs [173].

As mentioned previously, AD development may occur, inter alia, as a result of β-amyloid plaque formation [174]. The introduction of β- and γ-secretase inhibitors into clinical use could be an undisputed success in the treatment of AD. However, these drugs are not widely used in the treatment of AD due to their interaction with APP [175,176,177]. It is a participant in many neurotransmission reactions; therefore, despite the selective blocking of β- and γ-secretase and their effect on APP, we cannot exclude the possible side effects associated with the use of β- and γ-secretase inhibitors [175,178,179]. Additionally, in the case of the action on γ-secretase, pinitol has gained more and more attention. Pinitol, also known as NIC5-15, is a cyclic alcohol that, when taken in doses greater than 2.0 mg/day, reduces β-amyloid deposition, preventing plaque formation and the development of AD. However, determining the extent to which taking this compound is safe for patients requires many clinical trials [180,181]. Currently, work is underway to introduce an immune therapy for Alzheimer’s disease [182]. AD immunotherapy is based on the administration of a vaccine containing β-amyloid fragments, which is conjugated with an adjuvant, i.e., a substance whose main function is to enhance the immune response of the vaccine [183,184]. In this case, the immune response is based on the production of specific anti-Aβ antibodies, the presence of which prevents β-amyloid accumulation and formation. Currently, AD immunotherapy has been approved for phase I/II clinical trials [185].

In addition, immunotherapy for neuroinflammation in Alzheimer’s disease may involve stimulating the immune response with administered drugs. Moussa et al., conducted a study on the clinical utility of resveratrol as an agent to stimulate immune responses in patients, using a comparative analysis of a panel of markers in the plasma and cerebrospinal fluid (CSF) that included Eotaxin, TGF-α, IFNα2, IFNγ, GRO, IL-10, MCP-3, IL-12P40, MDC, IL-12P70, PDGF-AA, IL-13, PDGF-AB/BB, IL-15, sCD40L, IL-17A, IL-1RA, IL-1α, IL-9, IL-1β, IL-2, IL-3, IL-4, IL-5, IL-6, IL-7, IL-8, IP-10, TNFα, TNFβ [186]. The study showed a reduction in the number of important markers of neuroinflammation in CSF, including matrix metalloproteinase 9 (MMP9), which is responsible for the progression of neurodegenerative changes in the brain of AD patients by regulating the secretion of pro-inflammatory cytokines and free radicals that cause the induction of oxidative stress [186]. Reduced levels of MMP9 in CSF can be linked to the phenomenon of the decreased permeability of CNS barriers, which is associated with the reduced migration of leukocytes into the brain and, consequently, reduced levels of inflammatory factors in the brain of AD patients. This is an indisputable argument to continue work on drugs that modulate the immune response as components of the immune system represent an important turning point in the treatment of neuroinflammation in AD patients [186].

Clinical trials were also conducted on AD therapy, with the use of antihistamines, more specifically, latrepyridine [187]. It affects the structure and function of mitochondria and protects against the neuronal apoptosis caused by the accumulation of β-amyloid, and reduces the concentration of intracellular β-amyloid. The neuroprotective effects of latrepyridine are currently being investigated in vitro and in vivo [188,189]. In recent years, therapeutic possibilities in the field of clinical transplantation have gained more and more interest [190]. The treatment of Alzheimer’s disease is based on exchangeable plasma transfusions to clear it of circulating β-amyloid [191].

This method was especially useful for patients whose symptoms began to worsen over time, as it significantly slowed down the progression of the disease [192]. There have also been attempts to treat Alzheimer’s disease with insulin. The intranasal administration of insulin increases its concentration in the CNS, which contributes to the improvement of cognitive functions and memory [189]. Treating inflammatory processes in Alzheimer’s disease, such as treating AD itself, can take many different directions [192]. However, the goal of each chosen direction is the same: the aim is to find a drug that could have the greatest possible specificity in relation to the components of the inflammation process, e.g., inflammatory factors or inflammatory cells, thus enabling the suppression of neuroinflammation while maintaining the body’s ability to defend itself against pathogens, so that the body does not remain defenseless against damaging factors that disturb its homeostasis [193,194].

Kellar et al., conducted a study in which they determined the effects of 12 months of intranasal insulin therapy on inflammatory markers and immune cell function in AD. The cyclic administration of intranasal insulin decreased pro-inflammatory IL-6 and kept its levels stable [195]. The drug additionally increased the concentration of IFN-γ in the CNS, demonstrating a neuroprotective effect by stimulating cytotoxic T cells, whose function was to protect CNS from pathogens, as well as regulatory T cells, whose function was to extinguish the excessive inflammatory response that occurred in AD [195]. In addition, IFN-γ plays a supervisory role in the activation of microglia cells to perform their physiological functions while extinguishing the pathological and neurodegenerative effects of these cells on neural tissue [195]. Intranasal insulin administration increased eotaxin levels: an important marker of the inflammatory response in AD. In addition, intranasal insulin administration increased concentrations of anti-inflammatory cytokines such as IL-2, MDC and CCL22, which are implicated in the regulation of microglia function by reducing its activation. However, the authors point out that further studies of the mechanisms of action on inflammatory and immune mediators affected by intranasal insulin are needed, which only confirm its candidacy as an ideal drug for the treatment of neuroinflammation in AD [195].

One of the goals of the pharmacotherapy of inflammation in Alzheimer’s disease is to target inflammatory factors, including cytokines, including TNF-α. As one of the pro-inflammatory cytokines, TNF-α plays a very important role in the initiation and maintenance of the process of neuroinflammation, as well as in the physiological processes of remembering and learning [196,197]. It regulates the movement of the synapses and also stimulates native phagocytes in the CNS, i.e., microglial cells, to trigger an inflammatory response [198]. It is also an important cytokine at a time when the inflammatory response becomes chronic, as it continues to promote inflammatory responses by affecting microglial cells and the secretion of other inflammatory mediators, further impairing the brain’s cognitive function and, thereby, progressing Alzheimer’s disease [199]. Additionally, studies show that there is a clear relationship between β-amyloid plaque formation and elevated TNF-α levels found in the blood plasma and cerebrospinal fluid of AD patients [200]. Treatment with anti-TNF-monoclonal antibodies such as infliximab, adalimubab, and the recombinant etanercept fusion protein has been investigated in animal models in the context of Alzheimer’s disease. These experiments showed that the progress of neuronal destruction processes slowed down [201]. Moreover, the activity of the β-secretase enzyme, which is mainly responsible for the formation of β-amyloid deposits, was reduced, which is a key and important achievement in the therapy of patients suffering from Alzheimer’s disease [202]. Scientists are also trying to determine the clinical usefulness of TNF-α synthesis inhibitors, which include, inter alia, imide immunomodulatory drugs, including thalidomide [6]. Thalidomide is a drug that reacts with the 3 ‘terminus of the TNF-α template RNA, which impairs the synthesis of this cytokine [203]. Reducing the efficiency of the TNF-α synthesis process caused a decrease in its concentration, thus improving the function of neurons and nerve conduction and delaying the development of Alzheimer’s disease [204].

It has also been shown that thalidomide inhibits the activation of microglial cells and astrocytes, which are cell mediators of neuroinflammation [203,205]. In addition, thalidomide, compared to NSAIDs, is characterized by a more efficient crossing of the blood–brain barrier and, thus, a higher affinity for the CNS, which is another factor supporting the routine use of these drugs in the treatment of AD. A similar effect was also shown by the thalidomide derivative 3,6′-dithiothalidomide (3,6′-DT) [206].

The administration of 3,6′-DT reduces the concentration of TNF-α as well as impairing the synthesis of β-amyloid when deposited in the form of deposits due to a decrease in the secretion of amyloid APP. Similar to thalidomide, it inhibits the activation of CNS phagocytes and impairs the process of tau protein phosphorylation [207]. In addition to TNF-α, the treatment of Alzheimer’s disease can also involve the inhibition of other pro-inflammatory factors [27]. Interleukins, including pro-inflammatory IL-12 and IL-23, may be targets for the treatment of AD. IL-12 was responsible for activating inflammatory cells, including microglia and astrocytes, and stimulating these cells to produce inflammatory factors, e.g., IFN-γ, IL-23 [208]. Similar to IL-12, it exerts a promoting effect on the inflammatory response by inducing acute phase protein synthesis and recruiting an immune response with T lymphocytes. Anti-IL-12 and anti-IL-23 monoclonal antibodies, however, are still in clinical trials [209].

The clinical utility of cyclosporine and tacrolimus in the treatment of AD neuroinflammation is currently being investigated [210]. These are immunosuppressive drugs used mainly in transplantology in patients with the allogeneic transplantation of the liver, heart, and heart together with the lungs, pancreas or kidneys [211]. Both cyclosporine and tacrolimus have also been found in applications in dermatology in the treatment of atopic dermatitis [212]. In the case of AD, the light ones act on the messenger RNA of the amyloid precursor protein to reduce its expression [213]. Moreover, tacrolimus inhibits the secretion of pro-inflammatory cytokines, further stimulating the phagocytosis of β-amyloid deposits by microglial cells. Moreover, both cyclosporine and tacrolimus significantly slow down the progress of pathological biochemical histological changes, reducing memory deficits that appear with age and delaying the deterioration of cognitive functions [214].

Neuritis in AD can also be treated with metformin. It is a dimethyl biguanide derivative that has been used in medicine since 1957 as an oral antidiabetic drug in the treatment of type 2 diabetes, especially in obese or overweight patients. When used in elderly patients, it increases life expectancy and reduces cognitive impairment [215]. In addition, metformin has a positive effect on the mitochondria of nerve cells, inhibiting the process of their damage, thus extinguishing the inflammation developing on the basis of ROS activity and oxidative stress [216].

Another important drug from the perspective of inflammation therapy in AD is N-acetyl-5-methoxytryptamine, otherwise known as melatonin. It exhibits properties that model the activity of inflammatory pathways [217,218]. Melatonin inhibits pro-inflammatory processes by slowing down the release of NO nitric oxide, activating cyclooxygenase-2 and the formation of the NLR-P3 inflammosome, which is the structure of cytosolic oligomers and is induced to activate the inflammatory response. Additionally, melatonin delays the secretion of pro-inflammatory cytokines by CNS phagocytes, including astrocytes and microglial cells, and reduces the toxicity of β-amyloid by damaging its structure [218].

Inflammation in Alzheimer’s disease can be treated with antioxidants. Oxidative stress and the reactive oxygen species that cause it damage mitochondria and trigger an inflammatory process within them [219]. Antioxidants protect mitochondria against the effects of oxidative stress, including damage to two mitochondrial complexes: complex I and complex II. Those that are clinically useful in the treatment of AD neuritis include MitoQ, MitoTEMPO, MitoVitE, 4,5-dihydroxy-benzene-1,3-disulfonate (Tiron), astaxanthin [219].

Currently, however, non-steroidal anti-inflammatory drugs (NSAIDs) have the most established position in the treatment of inflammation in AD [220]. Especially clinically effective is the use of NSAIDs over a longer period of time, limiting the development of inflammatory Alzheimer’s disease over time, but the longer the drugs are taken, the more the disease is limited. Non-steroidal anti-inflammatory drugs may affect, inter alia, the cells involved in the inflammatory process, e.g., popular ibuprofen reduces the activation of CNS phagocytes, including microglia and astrocytes, and thus, they do not exhibit pro-inflammatory activity, which delays the development of AD [220]. Moreover, it influences the level of produced cytokines, reducing their concentration in the inflammatory focus. In addition to ibuprofen, the anti-inflammatory drugs used are diclofenac, S-flurbiprofen and indomethacin [221]. Moreover, all of them may affect the structure of β-amyloid, thus significantly reducing its concentration in both the cerebral cortex and the hippocampus [222]. In addition to affecting inflammatory cells and the level of β-amyloid by changing its spatial conformation, NSAIDs modulate the processing of the amyloid precursor protein, impairing the synthesis of β-amyloid at an early stage, primarily affecting the form of Aβ-42, which is considered to be the most responsible for the formation of plaques [223]. Some NSAIDs, including ibuprofen, may interfere with the activity of enzymes, including γ-secretase and α1-antichymotrypsin, resulting in no abnormalities in APP folding and a lack of toxic oligomers responsible for plaque formation [220].

Apart from γ-secretase and α1-antichymotrypsin, NSAIDs inhibit the activity of COX-1 and COX-2 cyclooxygenases catalyzing reactions, the products of which are the cause of the cognitive decline and memory impairment in the pathogenesis of AD [224]. It is worth adding, however, that the mechanisms by which NSAIDs act in the pathogenesis of AD are not fully understood, and the effects of some of them are not entirely satisfactory. Nevertheless, the use of non-steroidal anti-inflammatory drugs is an important element of AD therapy due to the beneficial inhibition of disease progression [225].

Natural alternatives to the pharmacotherapy of inflammation in AD are also currently being sought. The candidate for this title is, inter alia, curcumin [226,227]. It is an ingredient of turmeric, widely used in Indian cuisine. One of the many different properties of curcumin is its anti-inflammatory and antioxidant properties [228]. It affects, inter alia, the reduction in the concentration and activity of cytokines visible to the neuroinflammatory environment, including IL-1β, IL-6, IL-18, TNF-α, IFN-γ and chemotactic factors conditioning the migration of inflammatory cells, including monocytes and macrophages, astrocytes and microglia cells to the site of the ongoing inflammatory process [229]. The inhibition of the activity of cytokines and chemotactic factors takes place by influencing the transcription factors of inflammatory factors, e.g., activating protein-1 (AP-1) and nuclear factor kappa B (NF- B), thus blocking the processes of their synthesis [230].

Another of the benefits of curcumin is its pharmacodynamic properties [231]. Not only is it a substance that crosses the blood–brain barrier, but also, due to its good bioavailability in oral preparations, it allows therapeutic concentrations in the brain of patients with AD to be obtained [232]. Additionally, curcumin can affect changes that are related to cholinergic neurotransmission, which may improve cognitive functions and memory in patients with AD. The interest in curcumin is confirmed by the growing number of clinical trials conducted with turmeric [231]. Research has been conducted, inter alia, by specialists from Japan. Hishikawa et al., found that after treatment with curcumin in turmeric, the quality of life of AD patients receiving turmeric improved significantly. Additionally, it confirmed that the use of turmeric had a beneficial effect on the treatment of patients with Alzheimer’s disease, thus proving the clinical utility of curcumin in the treatment of AD [233].

## 6. Summary

There are many mechanisms for the formation of AD, and inflammation is one of them. It involves complex processes—including both complement components and inflammatory cells and substances produced by them, such as cytokines and chemokines. All this correlates with the occurrence of neurodegenerative processes and damage to neurons, which leads to the loss of their functionality and clearly affects the development of AD symptoms. Although much has been established about the inflammatory processes in the pathogenesis of AD, many aspects remain unexplained. Future research into neuroinflammation should further elucidate the nature of these processes and identify the mechanisms involved in the course of the disease. Similar strategies should be adopted when designing diagnostics and the pharmacotherapy of inflammation in AD. Methods are needed to visualize the inflammation of the nerves at an early stage of its development in the most specific way so that the development of inflammation and AD can be unequivocally confirmed. Current treatment improves the quality of life of patients without eliminating the causes of the disease. Many drugs and methods are still in the phase of clinical trials, and their admission to AD, in addition to practice, will be a turning point in treatment.

## Data Availability

Not applicable.
